# Behavioral and Emotional Dynamics of Two People Struggling to Reach Consensus about a Topic on Which They Disagree

**DOI:** 10.1371/journal.pone.0084608

**Published:** 2014-01-10

**Authors:** Levent Kurt, Katharina G. Kugler, Peter T. Coleman, Larry S. Liebovitch

**Affiliations:** 1 Division of Mathematics and Natural Sciences, Queens College, City University of New York, Queens, New York, United States of America; 2 Department of Psychology, Ludwig-Maximilians-Universität München, Munich, Germany; 3 Department of Organization and Leadership, International Center for Cooperation and Conflict Resolution, Columbia University Teachers College, New York, New York, United States of America; 4 Departments of Physics and Psychology, Dean Division of Mathematics and Natural Sciences, Queens College, City University of New York, Queens, New York, United States of America; Cinvestav-Merida, Mexico

## Abstract

We studied the behavioral and emotional dynamics displayed by two people trying to resolve a conflict. 59 groups of two people were asked to talk for 20 minutes to try to reach a consensus about a topic on which they disagreed. The topics were abortion, affirmative action, death penalty, and euthanasia. Behavior data were determined from audio recordings where each second of the conversation was assessed as proself, neutral, or prosocial. We determined the probability density function of the durations of time spent in each behavioral state. These durations were well fit by a stretched exponential distribution, 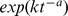 with an exponent, 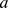, of approximately 0.3. This indicates that the switching between behavioral states is not a random Markov process, but one where the probability to switch behavioral states decreases with the time already spent in that behavioral state. The degree of this “memory” was stronger in those groups who did not reach a consensus and where the conflict grew more destructive than in those that did. Emotion data were measured by having each person listen to the audio recording and moving a computer mouse to recall their negative or positive emotional valence at each moment in the conversation. We used the Hurst rescaled range analysis and power spectrum to determine the correlations in the fluctuations of the emotional valence. The emotional valence was well described by a random walk whose increments were uncorrelated. Thus, the behavior data demonstrated a “memory” of the duration already spent in a behavioral state while the emotion data fluctuated as a random walk whose steps did not have a “memory” of previous steps. This work demonstrates that statistical analysis, more commonly used to analyze physical phenomena, can also shed interesting light on the dynamics of processes in social psychology and conflict management.

## Introduction

In all facets of life when individuals, groups, or nations interact with each other there is a possibility that a conflict can arise. In broad terms, a conflict is defined as different [1] or incompatible activities [2]. In detailed social psychological terms, a conflict is a relational process influenced by the presence of incompatible activities. These processes typically occur in a context that has a history and a normative trajectory. Conflicts perturb the flow of ongoing psychosocial processes [3].

Conflicts are often resolved in a fairly successful way. They can even lead to positive outcomes such as enhanced creativity and innovation [4],[5],[6]. However, a small fraction of conflicts intensify, escalate and persist, and thus become “intractable”. These intractable conflicts have severe consequences including: a wasting of time and money, a threat to psychological and physiological well-being, aggression, and even violence [7],[8]. They also have a high level of persistence, destructiveness [9], and resistance to resolution [10],[11],[7],[8],[12], [13], [14].

Understanding the dynamics underlying these intractable conflicts may help us to avoid or resolve them and therefore to prevent their potential negative impacts on our lives. For this purpose Kugler, Coleman and Fuchs [15] conducted a study (see [15], Study 1) where sets of two participants (dyads) were asked to discuss an intractable sociopolitical topic with another person, who disagreed on that topic. These “difficult conversations” represent a conflict created in the lab. Whereas some dyads were able to reach an elaborate common understanding of the conflictual topic and reported high levels of satisfaction with the discussion others did not reach a common understanding at all or a very poor understanding and reported low levels of satisfaction with the discussion. The outcomes of these extreme groups of discussions contain elements of intractable (those without or a poor a common understanding and low levels of satisfaction) and tractable (those with an elaborate common understanding and high levels of satisfaction) real world conflicts. We will refer to these extreme groups as “tractable” and “intractable” with the full understanding that they only represent a snapshot laboratory characterization in comparison to ongoing conflicts that are far less extreme and do not necessarily include all the characteristics present in their real world counterparts.

Comparing the conflict process of those extreme groups (i.e., tractable versus intractable discussions) offers an excellent possibility to learn about the psychological dynamics of tractable versus intractable conflicts. Kugler, Coleman, and Fuchs [15] found that more tractable conflicts evidenced high levels of complexity and openness in cognition, emotion and behavior of the participants. In contrast, more intractable conflicts displayed low levels of complexity and openness of these variables. The parameters for behaviors and emotions in that study reflected the dynamics of the entire conversation, as they were focused on averages across the course of the discussion.

These data may also provide an additional wealth of important information on the moment to moment dynamics. Therefore, in the present paper we use methods from statistical physics to analyze time series data from the described study. Using data on participants' behaviors and emotions over the entire course of the discussion we aim to determine how the values of those measures at one point in time are correlated with their values at previous points in time. In other words, we are interested in determining whether the behavioral and emotional states at one moment in time are effected by a “memory” of their previous states. The existence or degree of such memory may give us new insight into how these participants functioned in their difficult conversations. Such a “memory” could explain the perpetuating and enduring nature of intractable conflicts once a conflict becomes destructive.

In summary, we are interested in the underlying emotional and behavioral dynamics of conflict situations in general and tractable versus intractable conflict dynamics in particular. Whereas research has identified a large variety of different factors (related to the context, the issues, the outcomes, the relationships, and the processes) that constitute intractability [7], fundamental processes underlying intractable conflicts have rarely been studied [8], [9]. This paper contributes by exploring one aspect of the underlying dynamics: the “memory” in parties' emotions and behaviors. Thus we address the following two research questions: 1) Are the behaviors of people in a conflict influenced by their previous behaviors and are their emotions influenced by their previous emotions, that is, is there a “memory” in their behaviors or emotions? and 2) if there are such “memories” are they different in tractable versus intractable conflicts.

In order to answer these research questions we explore the behaviors and emotions of participants, who engaged in difficult dyadic conversations as described above. Participants' behaviors and emotions were coded throughout the entire difficult conversations. Kugler, Coleman and Fuchs [15] characterized behavior as “proself” (where personal goals dominate) or “prosocial” (where concerns for both the self and the other dominate). They also characterized emotion as the level of positive or negative emotional state. Research on conflicts has emphasized the role that these “proself”-versus-”prosocial” behaviors play in conflict management. More precisely prosocial motives were found to foster constructive conflict processes, and outcomes [15], [16]. However this research typically measures individual momentary motivations or long-term preferences using scales at one point in time. In our analysis we explore the change from proself to prosocial motives in conflicts over time. Similarly the experience of positive versus negative emotions in conflict situations has yielded important results. For example previous research has identified the central importance of understanding the ratio of positivity-to-negativity in predicting difficulties in social relations [15], [16], [17]. Positive and negative emotions are thought to build-up incrementally over time in relationships, affecting how specific encounters are experienced and interpreted. Rather than looking at ratios, we investigate the “memory” of emotions in conflict dynamics.

This paper is arranged as follows. First, it provides more details about the study conducted by Kugler et al. [15] and the emotion and behavior data obtained from it. Then it describes the statistical and mathematical methods we used to analyze the data and the results of that analysis. Finally, we summarize our findings and discuss their implications.

## Methods

### Participants and Procedure

The study (for more details see [15]) was conducted at Teachers College, Columbia University in the USA by recruiting 118 participants, who formed 59 dyads based on opposite views on a sociopolitical issue such as abortion, death penalty, euthanasia, or affirmative action, as determined by an initial pretest-questionnaire. After matching two participants with opposite views on one of these sociopolitical topics, they were asked to engage in a discussion on the issue for about twenty minutes and prepare a joint statement. The participants did not know a priori that they held opposite views on the subject they were discussing. The discussion was audio recorded.

The quality of the joint position statement was used to determine the degree of tractability of each conflict (i.e., outcome measures). Participants themselves listened to the discussion directly afterwards and coded their own emotional experience during the discussion (i.e., emotion data). Trained coders, who also listened to the discussions, coded participants' verbal utterances for a proself or a prosocial motivation (i.e., behavior data).

Among those who participated in the survey, 78% were female and 22% were male. The average age of all participants was 28.91 years with a standard deviation of 7.87 years. The highest educational achievement of the participants was that 1% had a high-school diploma, 70% a bachelors degree, 27% a masters degree, and 2% a Ph.D. Their ethnicity was 7% African-American, 19% Asian-American, 7% Latin-American, 54% White-American, and 13% other.

This research was conducted according to the principles expressed in the Declaration of Helsinki. Participants provided written agreement to the informed consent. First, in the online-pretest participants read the informed consent in the beginning of the questionnaire and were asked to indicate their agreement to participate in the study by checking a box. Second, during the session participants were given a printout of the informed consent and were asked to sign the consent if they agreed to participate. The study was approved by the Internal Review Board of Teachers College, Columbia University, New York, NY, USA.

### Outcome Measure and Selection of the Data Set

In the present analysis, we used data from only 23 dyads out of those 59 dyads that participated in the experiment. This is because we wanted to compare the two extreme groups, namely, those associated with either the most intractable or the most tractable outcomes of the conversation. Dyads were assigned to these extreme groups based on the joint statement that they wrote after the conversation. Each statement was assigned a level of degree of sophistication of political reasoning from 1 to 5 by trained coders [15]. Level 1 stands for poorly developed political reasoning and level 5 represents a very elaborated political reasoning. The 11 dyads whose statement was coded as level 1 or who were unable to write a joint statement at all were identified as the intractable dyads and the 12 dyads whose statement was coded as 4 or 5 were identified as the tractable dyads. It is often useful to analyze data with respect to a continuous variable. However, in our case we focused on the most extreme groups of dyads as we were interested in exploring and comparing the characteristics of emotional and behavioral dynamics of tractable versus intractable conflicts. We have no underlying model to suggest the functional form (linear or otherwise) of the dependence on that variable. For that reason we chose to dichotomize the groups into intractable and tractable to increase the likelihood that we could determine the characteristics that are different between those groups. This procedure also increases the sensitiity of detecting small differences in characteristics between these two groups.

### Behavior Data

From the audio recordings the behavior of each participant at each second was identified as proself, prosocial, or neutral [15],[18],[16]. Proself behaviors are those which focus on one's own goals and dictating one's own views to the other. These behaviors represent a competitive focus. On the other hand, prosocial behaviors are those which look for a common ground to compromise on the issue, and therefore reflect a cooperative focus. Whenever a participant is in neither behavior state, and therefore neutral, means either that person is in a listening mode or makes comments that have neither a proself nor a prosocial focus.

Trained coders listened to the audio recordings of the difficult conversations and coded the behaviors of each participant at each second as 1 for proself, 2 for neutral, and 3 for prosocial. This behavior data represents the way that each participant acted during the discussion and how he or she switched his or her focus in time. The data was coded using the computer program This “the mouse paradigm”, which was developed by Nowak and Vallacher [19] who successfully applied it to analyze the data from a number of different social psychology experiments. For more details on the coding process or the behavior data see [15].

### Emotion Data

Following the discussion, each participant was asked to listen to the audio recording and recall their emotions at each moment during the conversation. Using the “mouse paradigm” [19] they were instructed to move a computer mouse toward the left to indicate negative emotions (with very negative emotions at the far left of the computer screen) and toward the right to indicate positive emotions (with very positive emotions at the far right of the computer screen). The middle of the screen indicated neutral emotions. The position of the mouse was imaged along a horizontal axis on a computer screen. The integer values of the pixels, from zero (maximum negative emotional on the left) to 1123 (maximum positive value on the right) were recorded at each second. Neutral emotions were at the middle of this scale. The emotion data is represented by the time series of these pixel measurements. These data represent a measurement of the valence (positive versus negative) and the arousal (degree of positivity and negativity) of each participants own experience. Because participants coded the emotions themselves this measure assesses the valance and the arousal of participants' emotional experience independent from the reason for the emotional experience. For more details on the coding process or the behavior data see [15].

### Goals of the Analysis

The goal of the analysis is to directly address the two research questions. Our analysis of the data does not depend on choosing an a priori mathematical model. Rather we chose analysis methods (Probability Density Function, Hurst rescaled range, power spectral density) that reveal dynamical mathematical properties of the data without making any model assumptions. Our first research question is then to ask: what are the types of mathematical models that would produce those observed properties. This leads us to understand whether the observed behavior or emotion data could be produced (sufficient but not a necessary condition) by processes with or without a “memory” of past events and the explicit mathematical form of that memory. Understanding that memory is, or is not involved, and its specific form, is helpful to gaining further insight into behavior and emotion. However, since a very broad class of process can produce data with, or without, such memories of past events, we cannot, determine which specific process (or its associated specific mathematical model) underlies these behaviors or emotions. Our second research question is to explore whether those dynamics are different in tractable versus intractable conflicts.

### Differences between the Behavior and Emotion Data

The behavior data consists of discrete coded elements having the values of only 1, 2, or 3 that represent proself, neutral, or prosocial behavior. The emotion data consists of integers spanning a range of 0–1123 that form almost a continuous signal representing a range from very negative to very positive emotions with neutral emotions in the middle. Because of the difference between the behavior and emotion data we found it necessary to use different statistical methods to analyze each type of data. In order to analyze the behavior data we need to use mathematical methods best suited to studying data with only a few discrete levels. For this data we analyzed the durations of time spent in each of these three levels to determine the probability density function of the time spent in each behavioral state. That analysis provides information on the durations themselves and also on how the probability of switching between states depends on the duration of time already spent in a given state. In analyzing the emotion data we used methods for continuous signals, namely, the Hurst rescaled range analysis and power spectral density. These methods provide information about the time correlations in the data and its frequency components. (The number of durations of times spent in each of the three behavior states does not provide enough data for the Hurst rescale range or power spectral density analysis.) In addition we dichotomized the emotion data in order to apply the same procedures that were used for the behavior data. In the next sections, we describe these methods and the results that we found for both the intractable and tractable dyads.

## Analysis of the Behavior Data

### Frequency Histograms of the Time Durations in Behavior States

**Figure 1:**
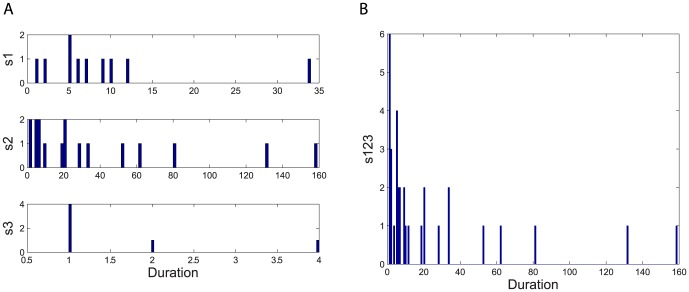
Frequency histograms of behavioral states for person 1 of intractable dyad 16. Histograms for the durations of time spent in the behavioral states: A) proself (s1), neutral (s2), prosocial (s3) and B) the combined states (s123) are shown as one example of how much time each participant spends in each of those behavioral states.

The durations of times spent in each of the three behavior states provides important information on both the durations of those states and on the probabilities of switching between those states. Thus, for each state, we first determined the number of seconds during which the behavior remained in that state. To analyze the behaviors of proself (state 1), neutral (state 2) and prosocial (state 3), we first constructed histograms of these durations for state 1 of each person, state 2 of each person, state 3 of each person and the combined states of 1, 2 and 3 (state 1+2+3) of each person. Next, separately for the intractable and tractable dyads, we combine state 1 of all dyads, state 2 of all dyads, state 3 of all dyads and finally state 1+2+3 of all dyads. As an example, Figure 1 shows frequency histograms of state 1 (s1), state 2 (s2), state 3 (s3) and combined states of all (s123) of person 1 in intractable dyad 16.

### Probability Density Function (PDF) of the Time Durations in Behavior States

Following the frequency histograms, the natural question is to ask whether these data sets have any specific distributions or not. There are a number of well-known distributions, each with their own specific mathematical form, that are generated by different types of mechanisms. The best known distribution is the “normal” or Gaussian distribution, but there are other distributions that are also found in experimental data. For example, a dataset that is a “fractal” [20], [21] has a frequency distribution that obeys power law, 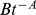, where the extreme values of that distribution, sometimes described as a “long tail” or “fat tail”, have a much higher probability of occurrence, than that expected of similar extreme values in a “normal” distribution.

Our aim here is to first construct the mathematical function called the probability density function, PDF, in the analysis of the intractable and tractable frequency distributions. The PDF is defined as the derivative of the cumulative distribution function, 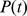,
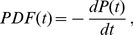
(1)where 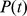 describes the probability that a value larger than 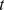 is found in the data.

It is important to note that there are not enough individual durations in each state for each participant to determine the PDFs separately for each participant. Therefore, we combined the data from each state for all the participants in either the intractable or tractable dyads to determine the PDFs from each state for the intractable or tractable dyads. As you will see, since the PDFs of each of these three states had a similar form, we also combined the data from all three states from all the intractable or tractable dyads to determine the PDFs of all three states combined for the intractable or tractable dyads.

A PDF is a curve and it may be obtained by smoothing a histogram, such as those shown in the previous section. The method we used here was to determine the PDF from the number of values 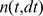 in the data that lie in the bin from 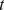 to 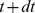 using histograms of different bin size 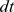 [22], [23]. This method improves the resolution at both the small and large values compared to computing the PDF from smoothing a single histogram with a fixed bin size. The points that form the PDF are then
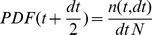
(2)where 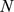 is the total number of data values [20]. We can now determine the functional form of the PDF, that is, whether the PDF is one of a number of common forms such as a single exponential, 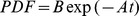; a power law 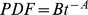; or a stretched exponential, 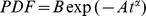.

**Figure 2:**
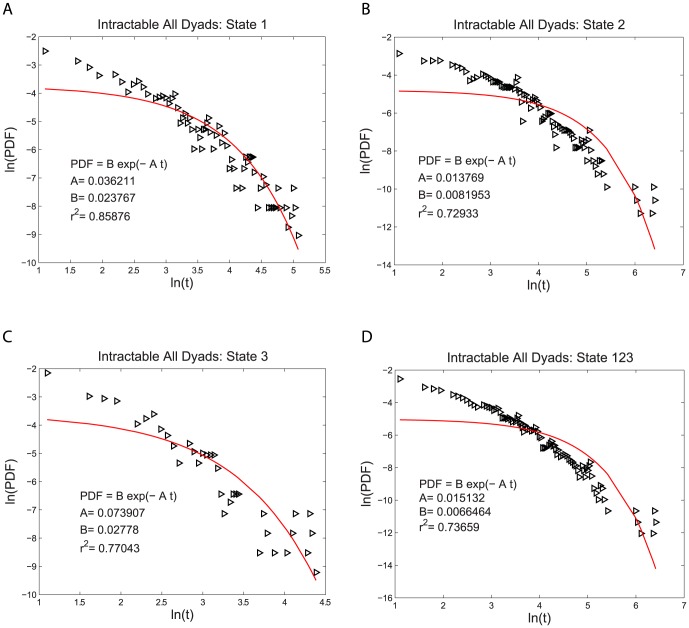
Single exponential probability density functions, PDFs, fit to the durations of the time, t, spent in each behavior state for the intractable dyads. The logarithmic plots of PDF versus duration time for A) proself (state 1), B) neutral (state 2), C) prosocial (state 3) and D) the combined data from all three states (state 123). The single exponential form is not a very good representation of this experimental data. The fitting parameters are given on each graph for each case.

**Figure 3:**
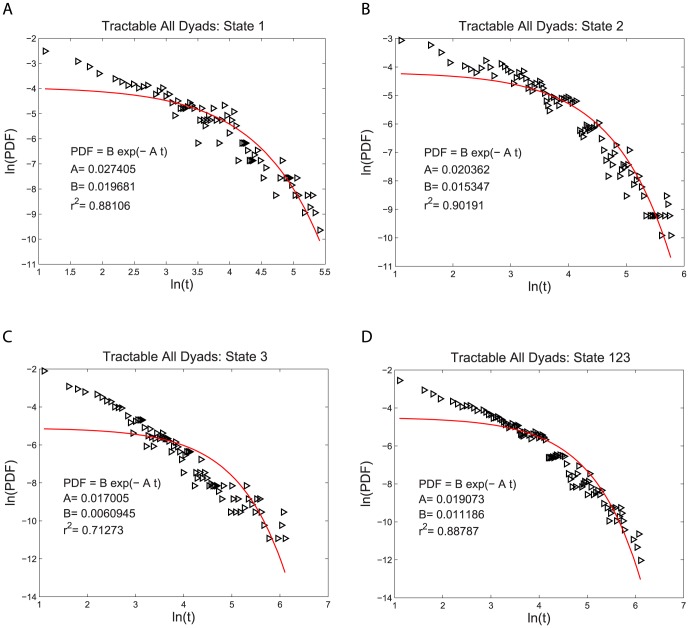
Single exponential probability density functions, PDFs, fit to the durations of the time, t, spent in each behavior state for the tractable dyads. The logarithmic plots of PDF versus duration time for A) proself (state 1), B) neutral (state 2), C) prosocial (state 3) and D) the combined data from all three states (state 123). The single exponential form is not a very good representation of this experimental data. The fitting parameters are given on each graph for each case.

**Figure 4:**
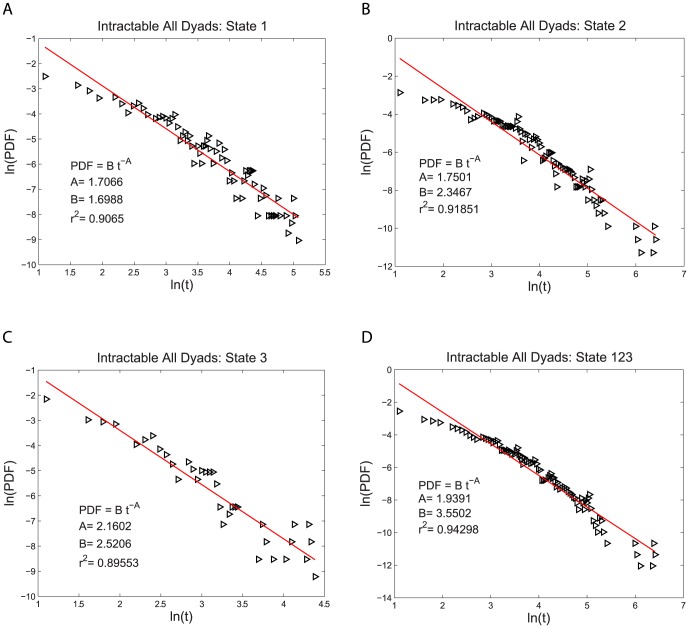
Power law probability density functions, PDFs, fit to the durations of the time, t, spent in each behavior state for the intractable dyads. The logarithmic plots of PDF versus duration time for A) proself (state 1), B) neutral (state 2), C) prosocial (state 3) and D) the combined data from all three states (state 123). The fit of the power law form is better than that of the single exponential form. The fitting parameters are given on each graph for each case.

**Figure 5:**
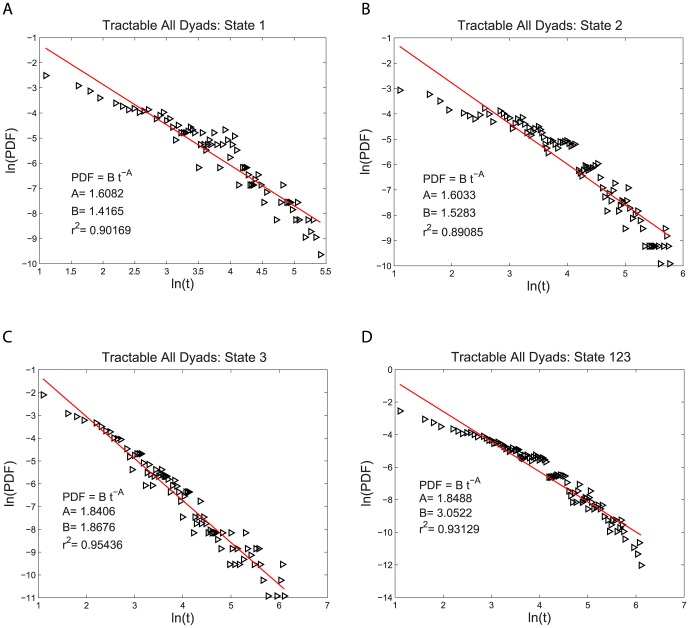
Power law probability density functions, PDFs, fit to the durations of the time, t, spent in each behavior state for the tractable dyads. The logarithmic plots of PDF versus duration time for A) proself (state 1), B) neutral (state 2), C) prosocial (state 3) and D) the combined data from all three states (state 123). The fit of the power law form is better than that of the single exponential form. The fitting parameters are given on each graph for each case.

**Figure 6:**
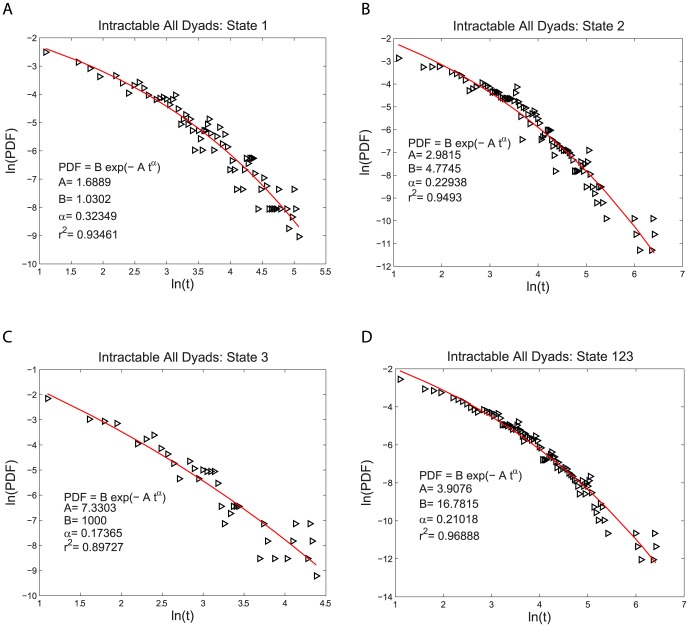
Stretched exponential probability density functions, PDFs, fit to the durations of the time, t, spent in each behavior state for the intractable dyads. The logarithmic plots of PDF versus duration time for A) proself (state 1), B) neutral (state 2), C) prosocial (state 3) and D) the combined data from all three states (state 123). The fit of the stretched exponential form is better than that of either the power law or the single exponential form. The fitting parameters are given on each graph for each case.

**Figure 7:**
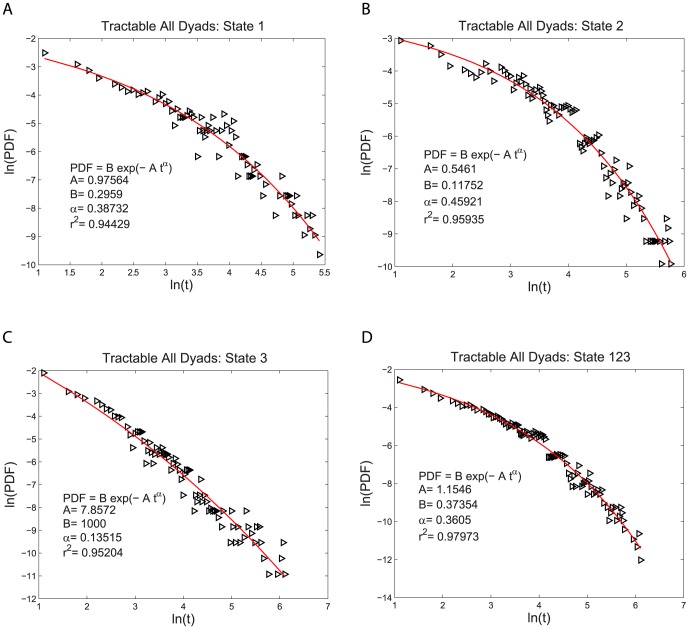
Stretched exponential probability density functions, PDFs, fit to the durations of the time, t, spent in each behavior state for the tractable dyads. The logarithmic plots of PDF versus duration time for A) proself (state 1), B) neutral (state 2), C) prosocial (state 3) and D) the combined data from all three states (state 123). The fit of the stretched exponential form is better than that of either the power law or the single exponential form. The fitting parameters are given on each graph for each case.

We used the method of least squares to find the best fit of each of these forms to the plot of 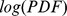 vs 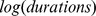 for both the intractable and tractable dyads and then compare their relative goodness of fit to the data. The three functional forms noted above have quite similar (nonlinear) shapes when plotted on linear plots of PDF(t) vs. t. For this reason we use plots of log PDF(t) vs. log (t) where the different shapes of these three functional forms can be more easily seen. This transformation, by itself, does not alter the quantitative analysis of the goodness of fit of these functional forms. The single exponential form is not a very good fit to the data, as shown in Figures 2 and 3. The power law form is a somewhat better fit to the data, as shown in Figures 4 and 5. The stretched exponential form appears to be the best fit of these three forms, as can be seen in Figures 6 and 7. That is, the PDFs of states 1, 2, and 3 separately and the combined data of states 1, 2, and 3, for both the intractable and tractable dyads are best represented by the stretched exponential form of
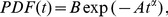
(3)where the averages of 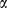 values of state 1, state 2, state 3 and state 123 are 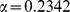 for the intractable dyads and 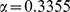 for the tractable dyads. This qualitative assessment of the goodness of fit is confirmed by values of the coefficient of determination 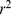 which are shown in tables 1 and 2. The closer the 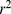 value to one, the better the fit. The 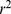 values are the largest for the stretched exponential form smaller for the power law form, and even smaller for the single exponential form for all the individual states as well as the combined states for both the intractable and tractable dyads (except that 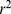 for state 3 of the tractable dyads is almost identical for both the stretched exponential and power law forms).

**Table 1:** Coefficient of Determination 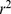 of the PDF Fits for the Intractable Dyads.

Intractable All Dyads	State 1	State 2	State 3	State 123
Stretched Exponential Fit	0.93461	0.94930	0.89727	0.96888
Single Exponential Fit	0.85876	0.72933	0.77043	0.73659
Power Law Fit	0.90650	0.91851	0.89553	0.94298

**Table 2:** Coefficient of Determination 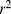 of the PDF Fits for the Tractable Dyads.

Tractable All Dyads	State 1	State 2	State 3	State 123
Stretched Exponential Fit	0.94429	0.95935	0.95204	0.97973
Single Exponential Fit	0.88106	0.90191	0.71273	0.88787
Power Law Fit	0.90169	0.89085	0.95436	0.93129

The PDF describes the distribution of the times spent in each behavior state. What is perhaps not obvious, is that it also reveals important information about the probability at each moment in time that the participant switches from one behavior state to another. That is because different types of probability to switch behavior states will then lead to different types of distributions of the times spent in each behavior state and therefore different types of PDFs. It is for this reason that we considered the single exponential, power law, and stretched exponential form of the PDF, each of which imply different types of the probability to switch behavior states. The fact that the stretched exponential form is the best fit to the PDF of the data reveals something quite interesting about the probability to switch behavior states. If there were a constant probability per second for a participant to switch from one behavior state to another, then the PDF would have a single exponential form. This is called a Markov process, in which the duration of times spent in the previous states, or even the duration of time already spent in the current state, do not effect when the switch occurs to a different state. That is, there is no “memory” of previous events in the dynamics of the system. This is clearly not the case. The better fit of the stretched exponential means that the probability to switch from one behavior state to another at each moment in time depends on the amount of time already spent in that state. The stretched exponential form means that the probability per second to switch states (as measured by the effective kinetic rate constant derived in Appendix S1) decreases with the duration of time already spent in the current state. Moreover, the PDF from the combined 1, 2, and 3 states of the intractable dyads has 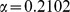, while that from the tractable dyads has 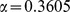. Smaller values of the parameter 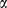 correspond to longer term memory. This implies that there is a longer term memory in the behavior of the dyads that were intractable compared to those that were tractable. However, since there is not enough individual durations in each state to determine the PDFs separately for each participant, we cannot estimate the variance in the 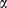 values between individuals and thus cannot determine if this difference in 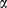 between the pooled data from all the individuals in the intractable and tractable dyads is statistically significant.

## Analysis of the Emotion Data

### Probability Density Function (PDF)

The emotion data consists of the set of pixels (from the most negative at 0 to the most positive at 1123), recorded each second by the participant moving a computer mouse to recall their emotion as they listened to the audio recording of the conversation. As it was done for the behavior data, we first estimated the PDF for this emotion data. However, we did not observe any consistent or familiar PDFs for the participants in either the intractable or tractable dyads.

### Hurst Rescaled Range Analysis

Long term correlations in the time series from many different phenomena have been analyzed by the rescaled range analysis (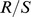 analysis) which was first introduced by Harold Edwin Hurst [24], [25], [26]. A key parameter in this analysis is to obtain the Hurst exponent, 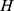, whose value provides important information about the correlations in time. When 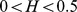 the time series is said to be “anti-persistent” meaning that positive fluctuations are more likely to followed by later negative fluctuations (and negative fluctuations by later positive fluctuations) at all time scales. When 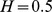 the fluctuations are uncorrelated as in ordinary Brownian motion. When 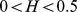 the time series is said to be “persistent”, meaning that positive fluctuations are more likely to followed by later positive fluctuations (and negative fluctuations by later negative fluctuations) at all time scales. Anti-persintence leads to more fluctuations in the time series, which are therefore rougher than the one generated by the ordinary Brownian motion, whereas persistence generates time series that are smoother than ordinary Brownian motion. To determine the Hurst exponent, the data is divided into 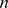 segments and the running sum of the values minus the average in that segment, divided by standard deviation in that segment is calculated. That value is called the rescaled range. The Hurst exponent is then computed as the slope of the best fit line on a plot of the logarithm of the rescaled ranges versus the logarithm of the size of the segments. In search of any correlations in the time series of emotions, we used the 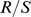 analysis to determine the Hurst exponent for each participant.

**Figure 8:**
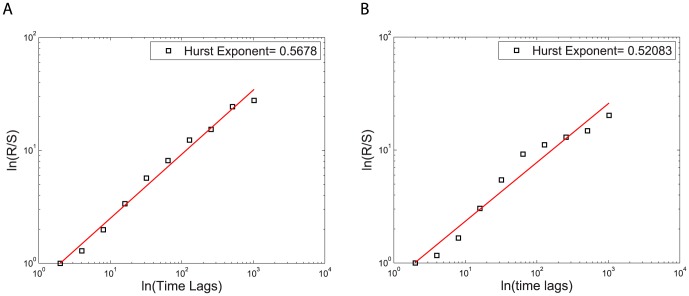
Hurst rescaled range analysis. The slope of the best straight line fit on the logarithmic plot of rescaled range (R/S) versus time is the Hurst exponent. The Hurst exponent 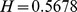 A) for person 2 in intractable dyad (A) and 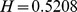 B) for person 2 in tractable dyad 10.

Figure 8 displays two examples of Hurst exponent plots, one for a person in an intractable dyad and one for a person in a tractable dyad. The data on these plots is approximately linear but shows some s-shaped curvature. This curvature was removed when the order of the pixel data was randomized indicating that this curvature contains information on higher order correlations within the data. Here we concentrate our analysis on those first order correlations. The averages of the Hurst exponents for both intractable and tractable dyads are slightly greater than 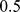 as seen in the tables 3 and 4. It is known that this method yields values of 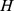 that are slightly greater than 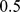 when the true value of 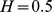 [27]. Values of 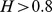 are typical of strong correlations in time. Hence the values of 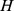 near 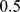 that we found from the emotion data are consistent with there being no strong time correlations in that data. We conclude that this emotion data is like ordinary Brownian motion with uncorrelated increments between the measured data values. This contrasts with the strong correlations in time that we found for the behavior data.

**Table 3:** Slope of the Logarithmic PSD Tail, Hurst Exponent and Coefficient of Determination 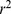 for the Intractable Dyads.

INTRACTABLE	Slope	Hurst Exponent	Coefficient of Determination 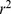
Dyad 16, P1	−2.0938	0.60361	0.98584
Dyad 16, P2			
Dyad 28, P1	−2.631	0.63296	0.97436
Dyad 28, P2	0.22497	0.54261	0.86969
Dyad 35, P1	−2.2759	0.63539	0.94833
Dyad 35, P2	−2.3371	0.57002	0.97129
Dyad 55, P1	−1.91031	0.51084	0.94802
Dyad 55, P2	−1.8448	0.55773	0.96837
Dyad 66, P1	−0.51172	0.47509	0.95689
Dyad 66, P2	−0.93651	0.56708	0.93265
Dyad 13, P1	−0.36911	0.60395	0.95947
Dyad 13, P2	−1.6332	0.50755	0.96727
Dyad 17, P1	−2.022	0.67403	0.97309
Dyad 17, P2	−1.9736	0.5678	0.98852
Dyad 40, P1	−1.9182	0.51084	0.94802
Dyad 40, P2	−1.8448	0.55773	0.96837
Dyad 45, P1	−2.082	0.56004	0.91230
Dyad 45, P2	−1.7389	0.54448	0.96898
Dyad 51, P1	−2.0174	0.50696	0.96593
Dyad 51, P2	−1.8904	0.64624	0.97262
Dyad 70, P1	−1.6154	0.56952	0.98387
Dyad 70, P2	−1.8859	0.67871	0.97001
**AVERAGES**	−1.6813	0.57253	0.95876

P1 and P2 in the first column represent person 1 and person 2. The results for dyad 16, person 2 are missing due to a technical reason involved in the experiment.

**Table 4:** Slope of the Logarithmic PSD Tail, Hurst Exponent and Coefficient of Determination 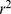 for the Tractable Dyads.

TRACTABLE	Slope	Hurst Exponent	Coefficient of Determination 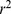
Dyad 10, P1	−1.7907	0.45568	0.95640
Dyad 10, P2	−1.907	0.52083	0.95754
Dyad 11, P1	−2.9231	0.54286	0.96659
Dyad 11, P2	−1.9741	0.55869	0.98584
Dyad 19, P1	−1.2818	0.38369	0.94559
Dyad 19, P2	−1.9186	0.46079	0.95028
Dyad 24, P1	−2.0536	0.63577	0.97202
Dyad 24, P2	−2.238	0.60131	0.93954
Dyad 26, P1	−1.8757	0.46605	0.93885
Dyad 26, P2	−1.6567	0.60522	0.99032
Dyad 27. P1	−2.1612	0.55534	0.95443
Dyad 27, P2	−1.8478	0.53232	0.97365
Dyad 34, P1	−1.3061	0.5282	0.98367
Dyad 34, P2	−2.5763	0.74248	0.96191
Dyad 57, P1	−1.7508	0.643	0.98540
Dyad 57, P2	−2.35	0.56284	0.96702
Dyad 58, P1	−1.6172	0.52584	0.97694
Dyad 58, P2			
Dyad 62, P1	−1.6357	0.6106	0.98635
Dyad 62, P2	−1.8098	0.61309	0.99112
Dyad 52, P1	−2.4083	0.66489	0.97331
Dyad 52, P2	−1.9335	0.48898	0.94049
Dyad 71, P1	−1.8385	0.5467	0.97723
Dyad 71, P2	−1.8338	0.60009	0.97709
**AVERAGES**	−1.942969565	0.558489565	0.96746

P1 and P2 in the first column represent person 1 and person 2. The results for dyad 58, person 2 are missing due to a technical reason involved in the experiment.

### Power Spectral Density and the Slopes of their Tails

We also determined the power spectral density, PSD, which makes it possible to look at signals in a frequency domain [28]. The signal's power is defined as the energy per unit time at each frequency. We determined the PSD using 4 methods: the FFT (Fast Fourier Transform) algorithm, peridogram, Welch PSD estimate, and Thompson multitaper PSD estimate, all of which yielded similar results. Frequency sampling was at 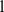 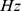 because the emotion data was sampled at one second in time. There were no frequency peaks observed in the PSD for any participant.

**Figure 9:**
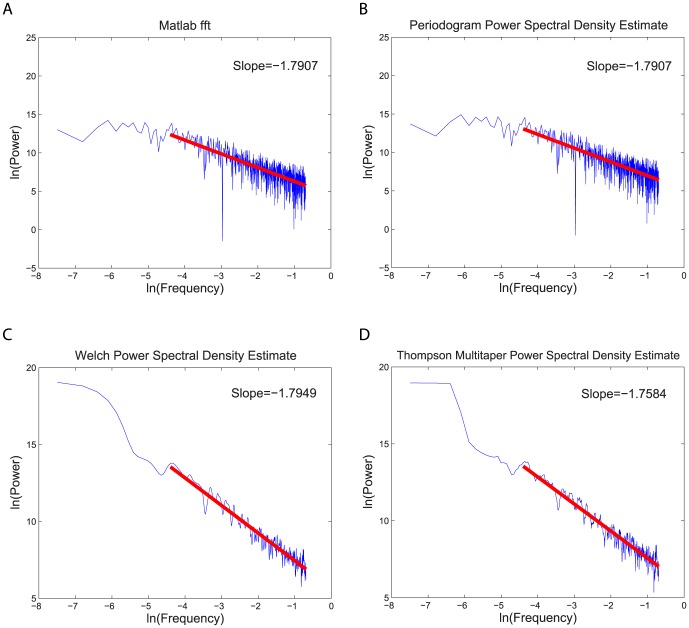
Power spectrum for person 1 in tractable dyad 10. The logarithmic graphs of power versus frequency are plotted using: A) an FFT algorithm, B) a periodogram power spectral density estimate, C) a Welch power spectral density estimate and D) a Thompson multitaper power spectral density estimate. The slope of the best straight line estimating the behavior of the tail is given on each graph. All four methods yielded similar values of the slope of the tail as given in the graphs.

Our main reason for determining PSD is that the linear slope of the tail of the PSD on a plot of the logarithm of the PSD vs. the logarithm of frequency is directly related to the Hurst exponent, so that the PSD can be used as an independent check on our determination of the Hurst exponent. The plots of of the natural logarithm of power versus the natural logarithm of frequency are shown in Figure 9. (These plots have more points at higher frequencies because the PSD is determined at linearly spaced frequency intervals.) We computed the slope of the tail in these plots. The four different methods produced almost the same values of the slopes from the data. This is also true for other data sets with the exception of a few isolated cases. Tables 3 and 4 list the slopes computed by periodogram method for each dyad (with rectangular windowing and neither zero-padding nor wrapping). The averages of these slopes are 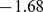 for the intractable dyads and 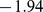 for the tractable dyads. We observed some abnormalities in a few of the power spectra. For instance, person 2 of intractable dyad 28 has a very different slope than all the others. If we ignore these unusual cases, the averages of the slopes are approximately 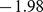 for the intractable dyads and 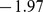 for the tractable dyads.

If the slope is determined from the data itself (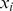) and the Hurst exponent is determined from the increments of the data (that is, 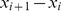), then the slope 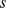 and Hurst exponent 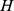 are related by 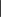
s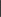 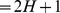. However, if both the slope 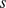 and the Hurst exponent are determined from the increments in the data, then the relation is 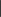
s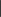 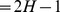 [26]. In our case, we computed the slope from the data and the Hurst exponent from the increments of the data. Since the average slopes are approximately equal to 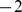, then we find that 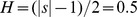. This result is consistent with the finding of the previous section that the Hurst rescaled range method shows that there are no correlations of the fluctuations in time in this data. The emotion data is well described by ordinary Brownian motion. The changes in the reported emotions at one moment in time have no dependence or memory of their changes at earlier moments in time.

### Mann-Whitney U Test of the Hurst Exponents between the Intractable and Tractable Dyads

We now investigate whether the quantitative measure of the time correlations in the emotion data, as determined by the Hurst exponent, 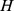, (or its equivalent logarithmic slope of the PSD) is statistically significantly different between the intractable and tractable dyads. In order to do this we use the rank-sum, non-parametric, Mann-Whitney U test. Such non-parametric tests do not require that the distribution of the values tested have an assumed form, such as that of a normal distribution, and therefore are more robust to different types of data [29]. We test the null hypothesis that the values of the Hurst exponent are the same for both the intractable and tractable dyads. From this method we found that that the one-sided p-value for the null hypothesis is 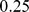. Values of 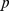 greater than the 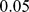 significance level indicate that we accept the null hypothesis. Therefore, we conclude that the Hurst exponent is the same for both the intractable and tractable dyads. We also performed the same statistical test on the coefficient of determination, 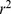, for the fit of a straight line on Hurst rescale range plots of the logarithm of the rescaled ranges versus the logarithm number of segments. This tests whether there is a difference in the nonlinearity of the time correlations between the intractable and tractable dyads. Here we found that the one-sided p-value yields 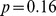 and so we also conclude that the nonlinearities of the time correlations are the same for the intractable and tractable dyads. Therefore, we found that there is no detectable difference in the fluctuations of emotion in time between the participants in the intractable and tractable dyads.

### Dichotomizing the Emotion Data to Compare it to the Behavior Data

Because of the differences between the behavior and emotion data we found it necessary to use different statistical methods to analyze each type of data. However, we can connect those two different analyses, to a degree, by dichotomizing the continuous emotion data so that we can better relate it to the dichotomous behavioral data. We determined the average value of the emotion data over a session and dichotomized it at each time point as 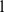 if it was below the average and 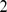 if it was above the average. The intervals of time spent in each of these two states are then the durations of time between crossing the average level. Unfortunately, this procedure produced relative few intervals (mean  =  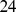) in each session and so the PDF cannot be reliably determined. However, the Hurst rescaled range analysis and the power spectral density analysis found that the change in the emotions of the participants were not correlated with their changes in emotion at previous moments in time. The PDF can however be calculated from a random walk which has exactly those properties. That PDF has the functional form proportional to 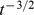 (see for example, the short time PDF distribution for a random walk on a finite 1-dimensional grid which corresponds to this case [30]). Such a random walk has a power law form, a straight line, on a plot of log (PDF) vs. log (t), which is significantly different than the stretched exponential form that is the best fit to the behavior data in figures 4 and 5. This further highlights the difference between the behavior and emotion data.

## Discussion

The study conducted by Kugler, Coleman and Fuchs [15] provide a unique laboratory setting to study how people behave in a conflict situation. These experiments consisted of two people (a dyad) engaging in a “difficult conversation” to prepare a joint statement on a sociopolitical and potentially intractable topic, on which they disagreed. Some of these dyads were unable to prepare a meaningful joint statement and thus represent a more intractable conflict. Others were successful in preparing a productive and elaborate, joint statement and thus represent a more tractable conflict. The behavior (namely the focus on proself versus prosocial motives) and emotion (namely the flow of emotions on a continuum from very positive to very negative with neutral in the middle) data from these experiments may help us to better understand the dynamics underlying intractable conflicts with deleterious outcomes or tractable conflicts which can be addressed in a positive way. In our analysis, we have reexamined this data to determine what it can also tell us about the dynamics, the changes in time, of the behavior and emotion of the participants during these conversations.

What does the new analysis presented here tell us, and what does it not tell us? As described below, these new results shed important new light on the dynamics of behavior and emotion over time. In particular they show the degree of influence of past behavior and emotion on future behavior and emotion. That is, the degree of “memory” in behavior and emotion. These new results do not predict the details of future behavior or emotions from past events, but they do provide an essential starting point in that any such predictive models must produce the type of memories observed in these analyses. Developing such predictive models could lead to a new understanding and therefore new methods of mitigating conflicts.

Summarizing the results of our analysis we will discuss the behavioral dynamics first before focusing on the emotional dynamics. We found that the probability density function, PDF, of the durations of times the participants spent in each of the proself, neutral, or prosocial behavior states, for both the tractable and intractable dyads, is not well fit by a single exponential form 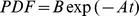. Such a single exponential form would be expected from a Markov process where the participants switch between behavior states with the same probability at each moment in time regardless of how long they have already remained in that behavior and regardless of their previous behaviors. On the contrary, we found evidence for non-Markovian behavior in that the data is better fit by a PDF with a stretched exponential form 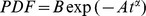. Many different phenomena, from the relaxation of spins in physical systems to the failure times of airplane parts, have a PDF distribution that is well fit by such a stretched exponential form (or its related Weibull distribution) [31], [32]. Typically this occurs in systems which have many pieces or processes that function in series or parallel which therefore generate a long term (nonlinear, non-exponential) memory that links future values to past values. The good fit of the stretched exponential PDF to both the intractable and tractable dyads implies that how the participants make decisions (conscious or unconscious) to shift their behavior is influenced by a “memory” of their previous behavior. The longer that they spend in one behavioral state, the less likely per second that they switch to another behavior state. Note that as 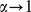 the stretched exponential distribution approaches that of a single exponential distribution with a strong (fast exponential) decay in the influence of past states on future states, while as 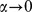 the stretched exponential distribution approaches that of a power law which has a very long time (slow algebraic) decay in the influence of past states on future states. The PDF from the combined 1, 2, and 3 states of the intractable dyads has 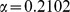, while that from the tractable dyads has 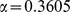. The smaller value of 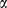 for the intractable dyads therefore suggests that there is a longer term memory in the behavior of the intractable dyads compared to tractable dyads. However, because of the need to pool data together across individuals in order to perform this analysis we cannot assign a reliable variance to these 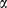
's and are therefore unable to determine if this difference is statistically significant.

We also found, using the Hurst rescaled range analysis and power spectral density that the change in the emotions of the participants was not correlated with their changes in emotion at previous moments in time. That is, the degree of negative or positive emotion was well described by a classical random Brownian motion. The emotional state fluctuated moment to moment without any “memory” of its previous fluctuations in time. A Mann-Whitney U test showed that there was no statistically significant difference between the intractable dyads and tractable dyads in this regard.

Reconnecting to our two research questions we can draw the following conclusions. Individuals' behaviors in the difficult conversations exhibit a notable “memory”, which means that once a specific behavior is shown, it will be more likely to be shown again. Kugler, Coleman, and Fuchs [15] found that for more intractable dyads proself motivated behaviors (where personal goals dominate) prevailed over prosocial motivated behaviors (where personal and common goals are balanced) while for more tractable dyads prosocial motivated behaviors prevailed over proself motivated behaviors. Therefore, the memory for behavior in the more intractable dyads reinforces their proself behaviors and increases their difficulty with preparing a joint statement, while the memory for behavior in the more tractable dyads reinforces their prosocial behavior and therefore increases their success in preparing a joint statement. In other words, once the conversation starts off with mainly proself motivated behaviors individuals will return to this behavior, resulting in a smaller and smaller likelihood that they will switch to prosocial motivated behaviors, which leads to a higher probability for intractable conflicts. Dyads, which start off with prosocial motivated behaviors are more likely to show these again and by focusing on common goals, thus create more elaborate common statements.

Even though these dynamics explain how intractable versus tractable conflicts evolve over time, there was not a large quantitative difference between those dyads: Both extreme groups - the intractable and the tractable dyads - showed a memory in the behavior data. However there was indeed a somewhat longer term “memory” in the behavior data of the intractable dyads compared to the tractable dyads, although this difference is not large. This small difference in longer term memory increases the influence of past behaviors, and so makes it even harder for the participants in these intractable dyads to successfully produce a joint statement. If this difference in the memory can be confirmed by future research, it might be an explanation for the enormous difficulty of transforming intractable conflicts toward more constructive and tractable dynamics [7],[8], [9].

Regarding the emotional experience of individuals involved in difficult conversations, we found the random fluctuations in emotion, which was surprising for us. In other words there was no memory in the emotional data. This pattern was the same for more intractable dyads and more tractable dyads. For our interpretation we combine those results with the analysis of Kugler, Coleman, and Fuchs [15]. Those authors found that for intractable dyads negative emotions prevailed over positive emotions while for tractable dyads positive emotions prevailed over negative emotions during the course of the entire difficult conversation. Thus, the fluctuations of emotion, as measured by the Hurst rescaled range analysis and power spectral density, were the same for both groups but there was a constant of emotion that was more negative for the intractable dyads and more positive for the tractable dyads. In other words, even though the emotions fluctuated randomly, they moved on average in different ranges on the scale from very positive to very negative emotions depending on whether the conflict was tractable or intractable.

Summing up, in tractable as well as intractable conflicts individuals' behavioral dynamics demonstrated a “memory” of the duration already spent in a behavioral state whereas their reported emotions changed in steps that did not evidence a “memory” of the previous steps. What are we to make of this difference between behaviors and emotions? The relationship between behavior and emotions is certainly complex and it would be very interesting for future research to examine this relationship in more detail. For example, looking at emotional ratings just before and just after the switches between proself, prosocial, and neutral behaviors is worthwhile and we would like to do that in subsequent studies, but that analysis is beyond the goals of this current study. Also emotions may vary as a function of the success or failure achieved in reaching a goal or can also be used in a negotiation in order to influence the behavior of another person, for example, in a negotiation. The relationship between emotional valence and pro-self versus pro-social behaviors can be complex. For example, positive valence could mean that a person is feeling good about their prosocial behavior or feeling good about their difficult or sarcastic proself behavior.

One possible interpretation of the results, that we present here to stimulate discussion on these issues, is that the changes in the emotional state of the participants, their changes in “feelings” were, to a degree, decoupled from their choices how to behave. This interpretation is supported by the work of Nowak and Vallacher [19] who proposed that social judgments involve a successive activation of many different cognitive and affective processes forming higher level decisions based on extensive neural computations. In physical systems, exactly such hierarchies of processes and/or multiple processes in parallel produce a PDF which is well fit by a stretched exponential form consistent with such a memory [31], [32]. On the other hand, emotional processes may function at a more basic lower level, fluctuating on a finer grained time scale, a stream of consciousness passing through the mind like the random walk of a dust grain buffeted first one way and then another by molecules of air. Certainly it would be very interesting to examine the relationship between emotions and behaviors in more detail.

Taken together, the analysis revealed interesting characteristics of underlying dynamics in conflicts. The memory, which we found for the behavioral dynamics, provides evidence for the perpetuating and enduring nature of intractable conflicts once a conflict becomes destructive. Therefore attempts to transform difficult and destructive conflict patterns might aim to constantly introducing positive interactions. Once the frequency of positive interactions increases it will be more likely that they are shown again in future. The puzzling fact that no memory for emotional dynamics was found might even be a chance for conflict resolution, as we could not find evidence for dynamics fostering an emotional deadlock in conflicts.

## Supporting Information

Appendix S1 Effective Kinetic Rate Constant.
(PDF)

## References

[1] Follett MP (1973) Power In Dynamic administration: The collected Papers of Mary Parker Follett. London, UK: Pitman.

[2] Deutsch M (1973) The resolution of conflict: Constructive and destructive processes. New Haven, CT: Yale University Press.

[3] Coleman PT, Kugler KG, Bui-Wrzosinska L, Nowak A, Vallacher R (2012) Getting down to basics: A situated model of conflict in social relations. Negotiation Journal 28: 7–43.

[4] De Dreu CKW (2008) The virtue and vice of workplace conflict: food for (pessimistic) thought. Journal of Organizational Behavior 29: 5–18.

[5] Tjosvold D (1998) Cooperative and competitive goal approach to conflict: Accomplishments and challenges. Applied Psychology: An International Review 47: 285–342.

[6] Tjosvold D (2008) The conflict-positive organization: it depends upon us. Journal of Organizational Behavior 29: 19–28.

[7] Coleman PT (2003) Characteristics of protracted, intractable conflict: Toward the development of a metaframework-i. Peace and Conflict: Journal of Peace Psychology 9: 1–37.

[8] Coleman PT (2006) Conflict, complexity, and change: A meta-framework for addressing protracted, intractable conflicts-iii. Peace and Conflict: Journal of Peace Psychology 12: 325–348.

[9] Vallacher RR, Coleman PT, Nowak A, Bui-Wrzosinska L, Liebovitch LS, et al.. (2013) Attracted to Conflict: Dynamic Foundations of Destructive Social Relations. New York: Springer.

[10] Bar-Tal D (2007) Sociopsychological foundations of intractable conflicts. American Behavioral Scientist 50: 1430–1453.

[11] Coleman PT (2000) Fostering ripeness in seemingly intractable conflict: An experimental study. International Journal of Conflict Management 11: 300–317.

[12] Kriesberg L (1999) Power In Dynamic administration: The collected Papers of Mary Parker Follett. New York, NY: Continuum, 332–342 pp.

[13] Kriesberg L (2005) Nature, Dynamics, and Phases of Intractability. Washington DC, USA: United States Institute of Peace. Grasping the Nettle. Analyzing Cases of Intractable Conflicts (pp. 65–97).

[14] Kriesberg L, Northrup T, Thorson S (1989) Intractable Conflicts and Their Transformation. Syracuse: Syracuse University Press.

[15] Kugler KG, Coleman PT, Fuchs AM (2011) Conflict, complexity, and openness: Constructive vs. destructive discussions on intractable issues. WOP Working Paper No. 2011/3. Retrieved from: www.psy.lmu.de/wirtschaftspsychologie/forschung/working papers/index.html.

[16] De Dreu CKW, Beersma B, Steinel W, Van Kleef GA (2007) Social psychology: Handbook of basic principles. New York, NY: Guilford Press.

[17] Gottman JM, Swanson C, Swanson K (2002) A general systems theory of marriage: nonlinear difference equation modeling of martial interaction. Personality and Social Psychology Review 6: 326–340.

[18] Losada M (1999) The complex dynamics of high performance teams. Mathematical and Computer Modelling 30: 179–192.

[19] Nowak A, Vallacher RR (1998) Dynamical Social Psychology. New York: The Guilford Press.

[20] Brown CT, Liebovitch LS (2010) Fractal Analysis. Thousand Oaks, CA: Sage.

[21] Liebovitch LS (1998) Fractals and Chaos: Simplified for the Life Sciences. New York: Oxford University Press.

[22] Liebovitch L, Sullivan J (1987) Fractal analysis of a voltage-dependent potassium channel from cultured mouse hippocampal neurons. Biophysical Journal 52/6: 979–988. 10.1016/S0006-3495(87)83290-3 PMC1330096 2447974

[23] Liebovitch L, Schwartz IB (2003) Information flow dynamics and timing patterns in the arrival of email viruses. Phys Rev E 68/1: 017101–1-017101-4. 10.1103/PhysRevE.68.017101 12935285

[24] Feder J (1988) Fractals (Physics of Solids and Liquids). New York: Springer.

[25] Mandelbrot BB (1982) The Fractal Geometry of Nature. New York: W. H. Freeman and Company.

[26] Churilla A, Gottschalke W, Liebovitch L, LY S, AT T, et al (1995) Membrane potential fluctuations of human t-lymphocytes have fractal characteristics of fractional brownian motion. Annals of Biomedical Engineering 24/1: 99–108. 10.1007/BF02770999 8669722

[27] van Beek J, Bassingthwaighte J (1992) Four methods to estimate the fractal dimension from self-affine signals (medical application). Engineering in Medicine and Biology Magazine, IEEE 11/2: 57–64. 10.1109/51.139038 PMC3459993 23024449

[28] Knight A (1999) Basics of MATLAB and Beyond. Chapman and Hall/CRC.

[29] Hollander M, Wolfe DA (1973) Nonparametric Statistical Methods. New York: John Wiley & Sons, Inc.

[30] Liebovitch L, Selector L, Kline R (1992) Statistical properties predicted by the ball and chain model of channel inactivation. Biophysical Journal 63: 1579–1585. 1283346 10.1016/S0006-3495(92)81732-0 PMC1262275

[31] Shlesinger MF (2001) Physics in the noise. Nature 411: 641. 11395745 10.1038/35079702

[32] Cox D (1970) Renewal Theory. London: Methuen & Co.

