# Supplementary material for: Behavioral and Emotional Dynamics of Two People Struggling to Reach Consensus about a Topic on Which They Disagree
Source: PLoS One. 2014 Jan 10;9(1):e84608. doi: 10.1371/journal.pone.0084608 (PMC3888402; doi:10.1371/journal.pone.0084608)
Supplement: Appendix S1 — Effective Kinetic Rate Constant. (PDF) [file pone.0084608.s001.pdf]

APPENDIX S1

Effective Kinetic Rate Constant

The probability of switching between behavioral states can also be described by the effective kinetic rate constant, k_{eff}, which is the probability to switch states given that the participant has already remained in that state for a time t. This parameter has proved valuable in analyzing a number of other different systems [1]. The effective kinetic rate constant has also been used in renewal theory, epidemiology and actuarial sciences. For example, k_{eff} in renewal theory, called age-specific failure rate, is the probability that a light bulb fails to produce light given that it has already produced light for t amount of time. In actuarial sciences, k_{eff} is called survival rate and used for life insurance policies. Therefore, we now derive that k_{eff} from the stretched exponential form used in the previous sections to show how our results here can be related to previous results found in other fields.

The escape probability k_{eff} is defined as [2]

$$k_{eff} = -\frac{d\,lnP(t)}{dt} \tag{1}$$

where $P(t)$ is the cumulative probability with the initial conditions of $P(t = 0) = 1$ and $P(t = \infty) = 0$. Once we carry out the derivative, we obtain

$$\frac{dP(t)}{P(t)} = -k_{eff}dt\,. \tag{2}$$

The probability density function (PDF) and $P(t)$ are related via

$$PDF = -\frac{dP(t)}{dt}\,. \tag{3}$$

As we have observed in fitting different forms of the PDFs to the experimental behavior data, the PDF for our data is well represented by the stretched exponential form

$$PDF = B\exp(-At^{\alpha})\,. \tag{4}$$

From equations (3) & (4), we get

$$B\exp(-At^{\alpha}) = -\frac{dP(t)}{dt} \tag{5}$$

$$dP(t) = -B\exp(-At^{\alpha})dt\,. \tag{6}$$

Integrating equation (6) from $t = 0$ to $t = t$ leads to

$$P(t) = 1 - B\int_0^t \exp(-At^{\alpha})dt\,. \tag{7}$$

The integration on the right hand side of the (7) is not trivial. Therefore, we solve the integral with the help of Maple software and find the analytical solution of (7) as

$$P(t) = 1 - BA^{-\frac{1}{\alpha}}k\left(\frac{\left(\alpha^2(\tau+1)+\alpha\right)\mathbf{M}\left(p,q,\tau\right)}{(1+\alpha)} + (1+\alpha)\mathbf{M}\left(p+1,q,\tau\right)\right) \tag{8}$$

with

$$p = \alpha^{-1} - \frac{1+\alpha}{2\alpha} \tag{9}$$

$$q = \frac{1+\alpha}{2\alpha} + \frac{1}{2} \tag{10}$$

$$\tau = At^{\alpha} \tag{11}$$

$$k = \alpha^2 t^{-\alpha+1} A^{\alpha^{-1}-1}\left(At^{\alpha}\right)^{-\frac{1+\alpha}{2\alpha}} \mathrm{e}^{-1/2\,At^{\alpha}}\,, \tag{12}$$

where $\mathbf{M}(p,q,\tau)$ is the Whittaker M function, which is related to the confluent hypergeometric function and arises as one of two linearly independent solutions to the Whittaker differential equation [3]. Since the solution is not a simple solution, we first check that the initial conditions for the cummulative probability (8) are satisfied, i.e., $P(t=0) = 1$ and $P(t=\infty) = 0$. The constant B in (7) or (8) is not an arbitrary constant, and can be found using the relation

$$\int_0^{\infty} PDF\,dt = 1\,. \tag{13}$$

Once we insert (4) in (13)

$$\int_0^{\infty} B\exp(-At^{\alpha})dt = 1\,. \tag{14}$$

we obtain

$$B = \frac{A^{\frac{1}{\alpha}}\alpha}{\Gamma(1/\alpha)}\,, \tag{15}$$

where $\Gamma(1/\alpha)$ is the gamma function and defined as [4]

$$\Gamma(x) = \int_0^\infty e^{-t}t^{x-1}dt\,. \tag{16}$$

Finally, we can summarize our results and put the effective kinetic rate constant in its final form. From equations (2) and (3), k_{eff} is given by

$$k_{eff}(t) = \frac{PDF(t)}{P(t)} \tag{17}$$

where

$$PDF(t) = B\exp(-At^\alpha)\,, \tag{18}$$

$$P(t) = 1 - BA^{-\frac{1}{\alpha}}k\left(\frac{\left(\alpha^2(\tau+1)+\alpha\right)\mathbf{M}\left(p,q,\tau\right)}{(1+\alpha)} + (1+\alpha)\mathbf{M}\left(p+1,q,\tau\right)\right) \tag{19}$$

with

$$\begin{aligned}
B &= \frac{A^{\frac{1}{\alpha}}\alpha}{\Gamma(1/\alpha)} && (20)\\
p &= \alpha^{-1} - \frac{1+\alpha}{2\alpha} && (21)\\
q &= \frac{1+\alpha}{2\alpha} + \frac{1}{2} && (22)\\
\tau &= At^\alpha && (23)\\
k &= \alpha^2 t^{-\alpha+1} A^{\alpha^{-1}-1} \left(At^\alpha\right)^{-\frac{1+\alpha}{2\alpha}} \mathrm{e}^{-1/2\,At^\alpha}\,. && (24)
\end{aligned}$$

To show why behaviors depend on the memory, let's first plot $ln(k_{eff})$ vs $ln(t)$ for both extreme groups. The values of A and α in calculating (8) are from the least square fits and they are displayed on Figures (6) and (7) in each case. In the following plots, B value is calculated using equation (15). As we see in Figures (S1) and (S2), k_{eff} decreases as time goes on. This means that the more time you spent in a state, the less probability there is to switch to another strategy, which implies a memory in the behavioral

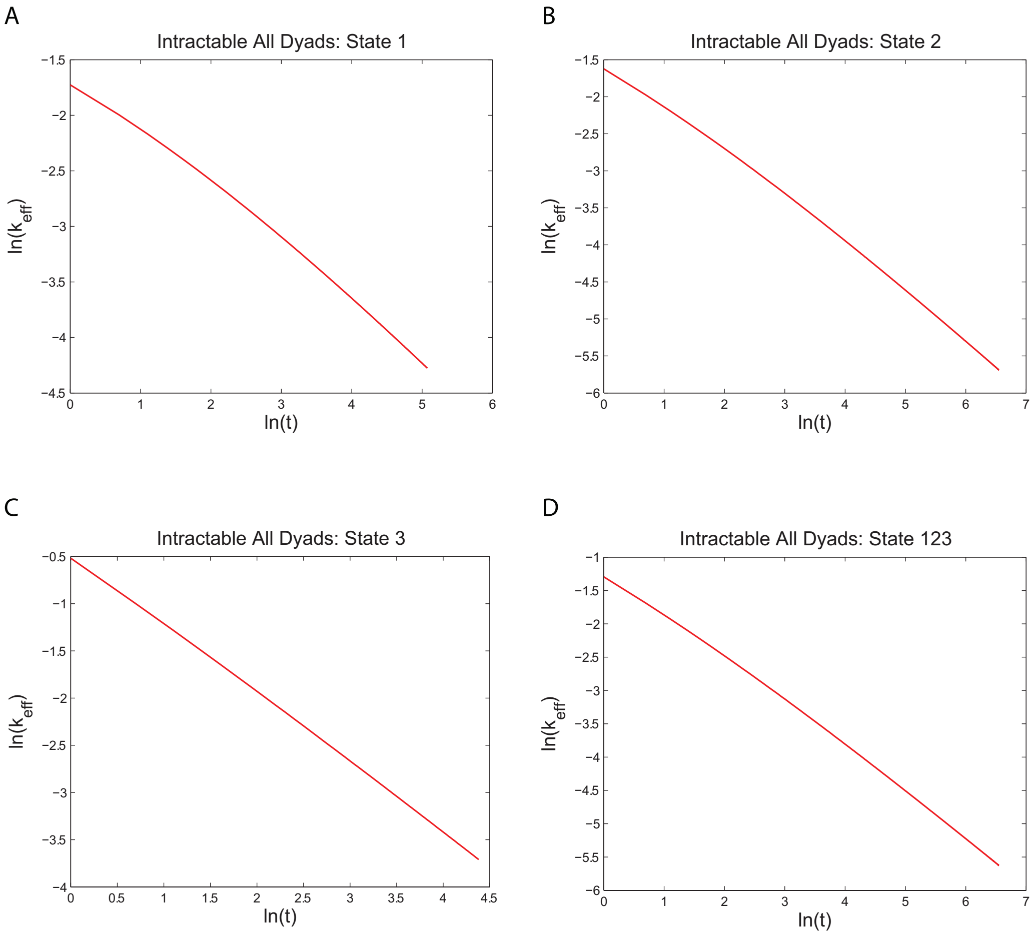

Figure S1. Plots of the effective rate constant for the intractable dyads. The logarithmic graphs of the effective rate constant versus time for all the intractable dyads for A) proself (state 1), B) neutral (state 2), C) prosocial (state 3) and D) the combined data from all three states (state 123). The plots indicate that k_{eff} is not a constant, as would be expected for a Markov process, but that it decreases with the time t already spent in a state.

dynamics.

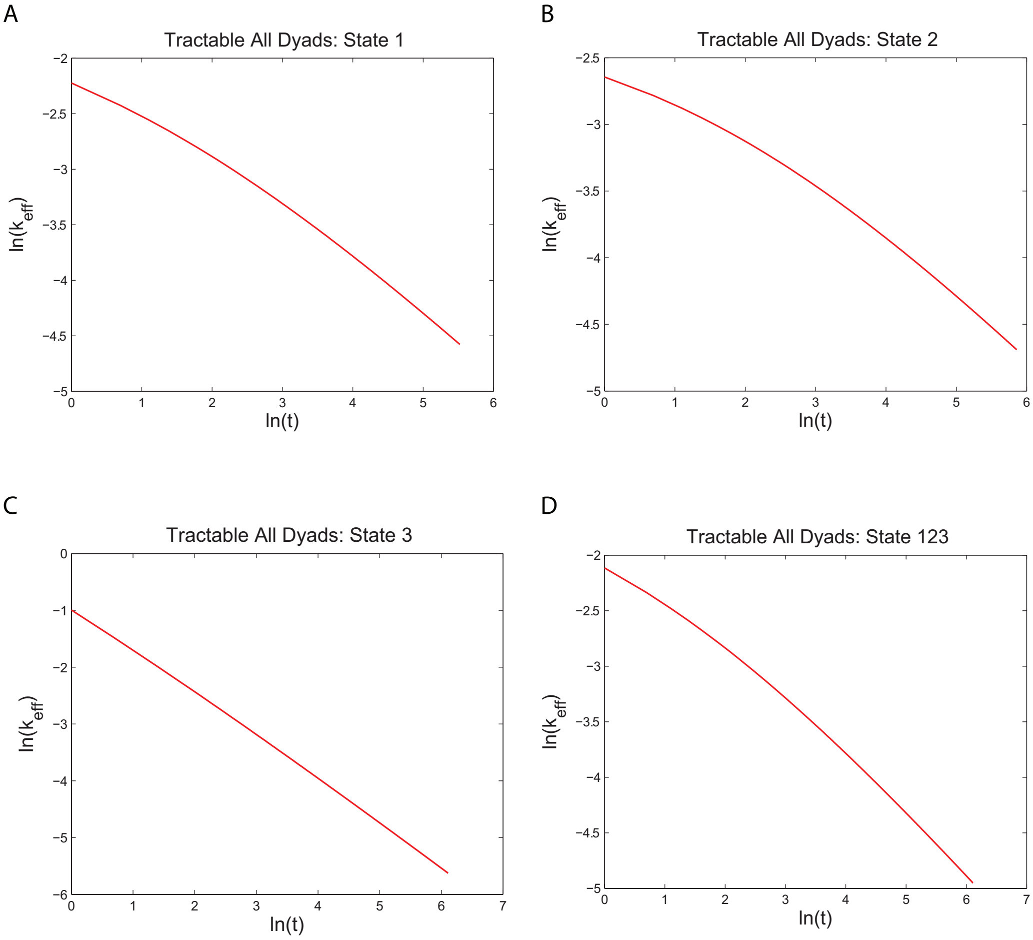

Figure S2. Plots of the effective rate constant for the tractable dyads. The logarithmic graphs of the effective rate constant versus time for all the tractable dyads for A) proself (state 1), B) neutral (state 2), C) prosocial (state 3) and D) the combined data from all three states (state 123). The plots indicate that k_{eff} is not a constant, as would be expected for a Markov process, but that it decreases with the time t already spent in a state.

Checking the Result for the Case $\alpha = 0.5$

To justify the general result we have obtained in the previous section, we will re-derive the k_{eff} (17) for the special case for $\alpha = 0.5$. We first get B using (15) as

$$\begin{aligned} B &= \frac{A^{\frac{1}{0.5}} 0.5}{\Gamma(1/0.5)} \\ &= \frac{A^2}{2} . \end{aligned} \tag{25}$$

The cumulative probability (7) reads as

$$P(t) = 1 - \frac{A^2}{2}\int_0^t \exp(-At^{1/2})dt\,. \tag{26}$$

Once we take the integral, the cummulative probability will be

$$P(t) = \mathrm{e}^{-A\sqrt{t}}\left(A\sqrt{t} + 1\right)\,. \tag{27}$$

One can easily check that $P(t = 0) = 1$ and $P(t = \infty) = 0$. This form of $P(t)$ looks much simpler than the one given in (8). To see the equivalence of (8) and (27) for the value of $\alpha = 0.5$, we compute and plot the cummulative probability using (8) and (27) for the parameters $A = 2$ and $t = 0.0001, 1.0001, 2.0001, ..., 25.0001$ using both of these two equations. We do not choose $t = 0$ because probability solution given by (8) does not converge at $t = 0$. However, as we make the time smaller and smaller towards zero, the cummulative probability approaches one for equation (8) as demanded by the initial conditions. Figure (S3) displays the $P(t)$ vs t graph and the differences between the two solutions

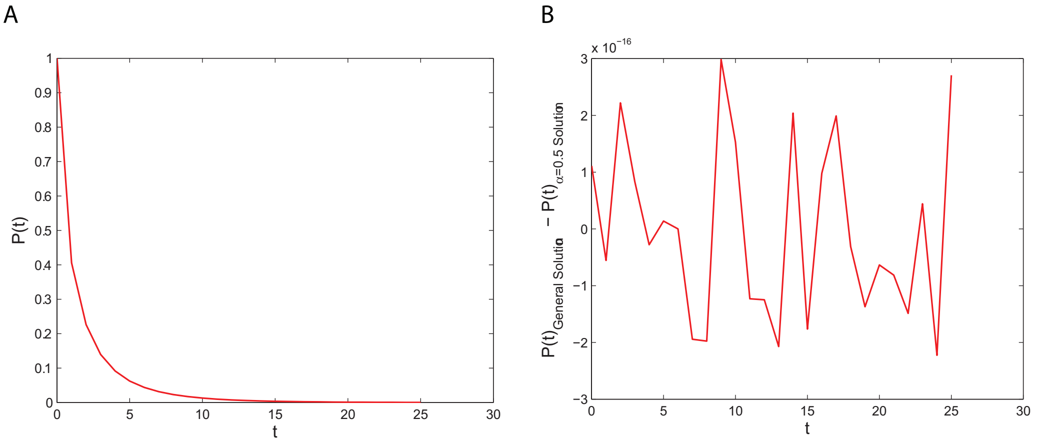

Figure S3. The cumulative probability graphs for $\alpha = 0.5$. On the left (A), the cumulative probability versus time graph is plotted by the simplified solution derived for $\alpha = 0.5$. On the right (B), the maximum difference between the general solution and simplified solution for the cummulative probability with α being 0.5 is in the order of 10^{-16}.

(8) and (27). It is clear that the two solutions generate almost identical results. Therefore, the general solutions for the escape probability (17) and the cummulative probability (8) based on the stretched

exponential form of the PDF are well justified.

References

1. Liebovitch L, Scheurle D, Rusek M, Zochowski M (2001) Fractal methods to analyze ion channel kinetics. Methods 24/4: 359-375.

2. Liebovitch L, Sullivan J (1987) Fractal analysis of a voltage-dependent potassium channel from cultured mouse hippocampal neurons. Biophysical Journal 52/6: 979-988.

3. Temme NM (1996) Special Functions: An Introduction to the Classical Functions of Mathematical Physics. New York: John Wiley & Sons, Inc., first edition.

4. Arfken GB, Weber HJ (1995) Mathematical Methods for Physicists. New York: Academic Press, fourth edition.
